# Fate of Transgenic DNA from Orally Administered Bt MON810 Maize and Effects on Immune Response and Growth in Pigs

**DOI:** 10.1371/journal.pone.0027177

**Published:** 2011-11-23

**Authors:** Maria C. Walsh, Stefan G. Buzoianu, Gillian E. Gardiner, Mary C. Rea, Eva Gelencsér, Anna Jánosi, Michelle M. Epstein, R. Paul Ross, Peadar G. Lawlor

**Affiliations:** 1 Teagasc, Pig Development Department, Animal and Grassland Research and Innovation Centre, Moorepark, Fermoy, Ireland; 2 Department of Chemical and Life Sciences, Waterford Institute of Technology, Waterford, Ireland; 3 Teagasc, Food Research Centre, Moorepark, Fermoy, Ireland; 4 Department of Biology, Central Food Research Institute, Budapest, Hungary; 5 Division of Immunobiology, Institute of Immunology, Medical University of Vienna, Vienna, Austria; Universidad Nacional Autonoma de Mexico, Instituto de Biotecnologia, Mexico

## Abstract

We assessed the effect of short-term feeding of genetically modified (GM: Bt MON810) maize on immune responses and growth in weanling pigs and determined the fate of the transgenic DNA and protein *in-vivo*. Pigs were fed a diet containing 38.9% GM or non-GM isogenic parent line maize for 31 days. We observed that IL-12 and IFNγ production from mitogenic stimulated peripheral blood mononuclear cells decreased (*P*<0.10) following 31 days of GM maize exposure. While Cry1Ab-specific IgG and IgA were not detected in the plasma of GM maize-fed pigs, the detection of the *cry1Ab* gene and protein was limited to the gastrointestinal digesta and was not found in the kidneys, liver, spleen, muscle, heart or blood. Feeding GM maize to weanling pigs had no effect on growth performance or body weight. IL-6 and IL-4 production from isolated splenocytes were increased (*P*<0.05) in response to feeding GM maize while the proportion of CD4^+^ T cells in the spleen decreased. In the ileum, the proportion of B cells and macrophages decreased while the proportion of CD4^+^ T cells increased in GM maize-fed pigs. IL-8 and IL-4 production from isolated intraepithelial and lamina propria lymphocytes were also increased (*P*<0.05) in response to feeding GM maize. In conclusion, there was no evidence of *cry1Ab* gene or protein translocation to the organs and blood of weaning pigs. The growth of pigs was not affected by feeding GM maize. Alterations in immune responses were detected; however, their biologic relevance is questionable.

## Introduction

Worldwide, the inclusion of genetically modified (GM) plants in animal feed and for human consumption has consistently increased over the past fifteen years since they were first cultivated in 1996 [1]. The increase in demand for GM ingredients has coincided with an 87-fold increase in cultivation area of GM crops reaching 148 million hectares worldwide in 2010 thus making the procurement of exclusively non-GM crops more difficult and expensive. In 2007, GM maize became the second most important biotech crop after GM soybeans [2] and the first one to have a wider variety of genetic modifications than glyphosate-tolerant soybean.

GM plants are designed to provide more nutritious food and to enhance agronomic productivity without the use of pesticides [3], [4]. However, the increased usage of GM crops for direct human consumption and feeding to meat- and milk-producing animals has lead to public concern. Consumer concerns are mostly related to a perceived risk to health, allergenicity of the transgenic proteins or the transfer of recombinant DNA from feed to livestock and livestock derived products that are consumed by humans [5]. Other concerns are associated with environmental issues such as gene transfer from GM crops to indigenous plants, reducing biodiversity and influence of the GM crops on non-target species [6], [7], [8], [9]. Adoption of GM technology has received varying degrees of support worldwide. However, much greater resistance to food biotechnology exists in Europe compared to other parts of the world [10].

The entry of GM plants into the food chain is highly regulated, particularly within the European Union where rigorous pre-market risk assessment is untaken to ensure the safety of GM plants for animal and human consumption. Numerous animal studies have focused on evaluating the risks of feeding Bt maize on health and growth parameters and no abnormalities have been identified [3], [11], [12], [13], [14], [15], [16]. However, some studies have found alterations in the immune response of mice fed Bt maize [17] and peas expressing the bean α-amylase inhibitor [18]. To date, the Cry1Ab protein has been proven safe in most animal studies. The transgenic protein has no homology to any allergenic proteins and was successfully degraded in simulated gastric conditions [19].

To fully address safety concerns related to GM feed ingredients, studies to determine the fate of ingested recombinant DNA fragments in animals have also been conducted. Many of these animal studies have failed to observe translocation of recombinant DNA fragments outside the GIT [20], [21], [22] although in some studies, low levels of recombinant DNA have been documented in the organs of pigs [23], [24].

The objectives of the experiments outlined in this paper were to evaluate both the intestinal and peripheral immune response in pigs in response to short-term GM maize exposure previously only conducted in mice. A further objective was to determine the fate of ingested recombinant DNA and protein in pigs thus allowing a clearer assessment of the safety of GM maize to be made.

## Methods

### 1. Ethics statement

The pig experiments described below complied with European Union Council Directive 91/630/EEC (outlines minimum standards for the protection of pigs) and European Union Council Directives 98/58/EC (concerns the protection of animals kept for farming purposes) and was approved by, and a license obtained from, the Irish Department of Health and Children (licence number B100/4147). Ethical approval was obtained from the Teagasc and Waterford Institute of Technology ethics committees.

### 2. Genetically modified maize

Seeds derived from MON810 and its parental control maize (PR34N44 and PR34N43 varieties, respectively: Pioneer Hi-Bred, Sevilla, Spain) were grown simultaneously side by side in Valtierra, Navarra, Spain by independent tillage farmers. The GM and isogenic control maize were purchased by the authors from the tillage farmers for use in this animal study.

### 3. Animal housing, diets and management

Two experiments were conducted to assess the effect of short-term feeding of Bt (MON810) maize on the peripheral and systemic immune response in weanling pigs and to determine the fate of transgenic DNA *in-vivo*.

#### 3.1 Expt.1

Thirty-two crossbred (Large White×Landrace) weanling pigs (entire males) were weaned at approximately 28 days of age and were blocked by weight and litter, and randomly assigned to one of two experimental treatments; (1) non-GM isogenic parent line of maize (Pioneer PR34N43) and (2) GM maize (Pioneer PR34N44 event MON810). A non-GM starter diet was fed *ad libitum* for the first 6 days post-weaning during an acclimatization period and either the non-GM or GM maize experimental diets were fed for the remaining 31 days. Diets were manufactured in the Moorepark feed mill and were formulated to meet or exceed the NRC [25] requirements for weanling pigs (Table 1). Stringent quality control measures were employed to avoid cross contamination of non-GM with GM diets. Carryover in the feed manufacturing system was minimized by flushing the system with non-GM ingredients and the preparation of non-GM diets prior to diets containing the GM maize. In addition non-GM soybean meal was used in the manufacture of all diets. Cereals were ground by hammer mill through a 3 mm screen before mixing. Diets were pelleted to 5 mm diameter after steam conditioning to 50–55°C. The GM and non-GM maize were tested for the presence of the *cry1Ab* gene, pesticide contaminants and mycotoxins as described by Walsh *et al.* [26]. Proximate, (FBA Laboratories, Waterford, Ireland) amino acid and carbohydrate analysis (Sciantec Analytical Services Ltd., Cawood, UK) of experimental diets was performed (Table 1).

**Table 1:** Composition of acclimatization starter diet and experimental diets (as is basis, %)^1^ ^2^.

	Expt. 1			Expt. 2			
	Baseline (day -6 to 0)	Experimental (day 0 to 31)		Experimental (Starter) (day 0 to 7)		Experimental (Link) (day 7 to 35)	
Ingredient, %	Non-GM	Non-GM	GM	Non-GM	GM	Non-GM	GM
Maize (non-GM)	27.33	38.88	---	27.33	---	38.88	---
Maize (GM – MON810)	---	---	38.88	---	27.33	---	38.88
Soya Hi-Pro (non-GM)	24.00	25.00	25.00	24.00	24.00	25.00	25.00
Lactofeed 70^3^	25.00	20.00	20.00	25.00	25.00	20.00	20.00
Immunopro 35^4^	12.50	9.00	9.00	12.50	12.50	9.00	9.00
Fat, soya oil	8.00	4.00	4.00	8.00	8.00	4.00	4.00
Lysine HCl (78.8)	0.30	0.30	0.30	0.30	0.30	0.30	0.30
DL-Methionine	0.25	0.20	0.20	0.25	0.25	0.25	0.25
L-Threonine (98)	0.12	0.12	0.12	0.12	0.12	0.12	0.12
L-Tryptophan	0.10	0.10	0.10	0.10	0.10	0.10	0.10
Premix^5^	0.30	0.30	0.30	0.30	0.30	0.30	0.30
Mycosorb^6^	0.20	0.20	0.20	0.20	0.20	0.20	0.20
Salt	0.30	0.30	0.30	0.30	0.30	0.30	0.30
Dicalcium Phosphate	0.50	0.50	0.50	0.50	0.50	0.50	0.50
Limestone flour	1.10	1.10	1.10	1.10	1.10	1.10	1.10
Analyzed Chemical Composition (%)							
Dry matter	91.3	89.4	89.2	91.7	91.3	91.3	90.9
Crude protein	20.9	20.9	21.1	20.5	21.8	21.1	20.1
Fat	9.6	6.1	5.9	9.8	9.8	7.1	6.7
Crude fiber	1.7	2.1	1.9	1.6	1.5	1.6	1.5
Ash	6.3	5.5	5.6	5.8	6.4	5.7	5.8
Lysine	1.55	1.42	1.42	1.48	1.39	1.43	1.50
Ca^7^	0.83	0.78	0.78	0.83	0.83	0.78	0.78
P^7^	0.61	0.59	0.59	0.61	0.61	0.59	0.59
Digestible energy, MJ/kg^7^	16.33	15.38	15.38	16.33	16.33	15.38	15.38

1 Non-GM starter diet fed to pigs (Expt. 1) for 6 days post-weaning.

2 Experimental GM and non-GM maize diets fed to pigs in Expt. 1 for 31 days and Expt. 2 for 35 days.

3 Lactofeed 70 contains 70% lactose, 11.5% protein, 0.5% oil, 7.5% ash and 0.5% fibre (Volac, Cambridge, UK).

4 Immunopro 25 is a whey protein powder product containing 35% protein (Volac, Cambridge, UK).

5 Premix provided per kg of complete diet: Cu, 155 mg; Fe, 90 mg; Mn, 47 mg; Zn, 120 mg, I, 0.6 mg; Se, 0.3 mg; vitamin A, 6000 IU; vitamin D_3,_ 1000 IU; vitamin E, 100 IU; vitamin K, 4 mg; vitamin B_12,_ 15 µg; riboflavin, 2 mg; nicotinic acid, 12 mg; pantothenic acid, 10 mg; choline chloride, 250 mg; vitamin B_1,_ 2 mg; vitamin B_6,_ 3 mg; and endox, 60 mg.

6 Mycosorb is organic mycotoxin absorbent (Allech Inc. Dunboyne, Ireland).

7 Calculated values.

Pigs were housed individually in a total of four rooms with eight pigs per room (16 pigs/treatment). Pigs were individually penned in fully slatted pens (1.07 m×0.6 m) with plastic slats (Faroex, Manitoba, Canada). Pigs had unlimited access to water and feed through a single bowl drinker fitted in each pen and a door-mounted stainless steel feed trough (410 mm long) with centre divider, respectively. Heat was provided by a wall mounted thermostatically controlled electric bar heater (Irish Dairy Services, Portlaoise, Ireland). The rooms were naturally ventilated with an air inlet in the door and exhaust by way of a roof mounted chimney. Temperature was maintained at 28–30^o^C in the first week and reduced by 2^o^C per week to 22^o^C in the fourth week. Lighting was provided by tubular fluorescent lights from 0830 h to 1630 h. Pigs were observed closely at least three times daily. Any pigs showing signs of ill health were treated as appropriate.

#### 3.2 Expt.2

A second experiment was conducted to examine the effects of short-term exposure to GM maize on local immune response of weanling pigs. Pigs (n = 20) were weaned at approximately 28 days of age and were blocked by weight and litter, and randomly assigned to one of two experimental treatments; (1) non-GM isogenic parent line of maize (Pioneer PR34N43) and (2) GM maize (Pioneer PR34N44 event MON810) similar to pigs in Expt. 1. Pigs were fed experimental starter diets from day 0 to 7 post-weaning and experimental link diets from day 7 to 35 post-weaning (Table 1). Pigs were penned individually in the same room for the duration of the experiment (35 days). Pens were fully slatted (1.2 m×0.9 m) with plastic slats (Faroex, Manitoba, Canada) and plastic dividers between pens. Water was available *ad libitum* from one nipple-in-bowl drinkers (BALP, Charleville-Mezieres, Cedex, France) per pen. Feed was also available *ad libitum* from a single stainless steel feeder 30 cm wide (O'Donovan Engineering, Coachford, Co. Cork). Environmental condition control and management of pigs was conducted in a same manner as outlined for Expt. 1.

### 4. Intestinal, organ and blood sampling

#### Expt. 1

On day 31, 10 pigs/treatment were sacrificed by captive bolt stunning followed by exsanguination. The last meal was administered 3 h prior to sacrifice. During the sampling procedure, special care was taken to prevent any cross contamination between the GM and non-GM maize-fed pigs. All non-GM maize-fed pigs were sacrificed first followed by the GM maize-fed pigs. All surgical instruments were cleaned with a 70% ethanol solution between each animal. During the sampling procedure, all assistants wore single-use gloves that were replaced after each sample was taken. The heart, liver, spleen, kidneys and a sample of the semi-tendinosus muscle were removed first, to prevent contamination with digesta contents, followed by the entire GIT. Whole blood samples were taken from the anterior vena cava of 10 pigs per treatment and collected in heparinised blood collection tubes (BD Vacutainer Systems, Franklin Lakes, NJ) on day 0 and 29. Samples were stored at room temperature and peripheral blood mononuclear cells (PBMC) were isolated and assayed within 30 h. Blood samples were also taken at slaughter (day 31) and collected in EDTA-containing tubes (BD Vacutainer Systems) and immediately placed on ice for transport to the laboratory. Blood samples were centrifuged at 2500×g for 20 min after which the buffy coat of white blood cells was removed and stored at −20^o^C for subsequent analysis for the *cry1Ab* gene. Plasma from these samples was stored at −20^o^C for subsequent Cry1Ab-specific Ig analysis. The heart, liver, kidneys and spleen were removed, trimmed of any superficial fat or blood clots. The outermost layer of each tissue was removed to ensure that samples were taken from interior sections to prevent any residues of feed causing contamination of the samples. Samples were taken from the liver (distal end centre of central lobe), kidney (middle of the kidney cortex and medulla), spleen (anterior end of spleen), heart (left ventricle wall) and semi-tendinosus muscle and snap frozen in liquid N and stored at −20^o^C for subsequent analysis of the Cry1Ab protein and gene. Digesta samples from the stomach, ileum, cecum and cecal samples were stored at −20^o^C for subsequent analysis of the *cry1Ab* gene and protein.

#### Expt. 2

On day 35, 10 pigs/treatment were sacrificed (as outlined in Expt. 1) and spleen samples were taken (anterior end) and placed on ice in Hank's balanced salt solution (HBSS; Sigma-Aldrich, St. Louis, MO) for splenocyte isolation. Ileal samples (15 cm distal to the ileo-cecal junction) were taken and placed on ice in HBSS (Sigma-Aldrich) for subsequent lamina propria and intraepithelial lymphocyte isolation.

### 5. Growth

#### Expt. 2

Individual body weight and feed disappearance were recorded on day 0, and 30 of the experiment for calculation of growth performance.

### 6. Evaluation of the immune response to oral administration of Bt MON810 maize in pigs

#### 6.1 Isolation and stimulation of PBMC and cytokine measurement

Isolation of PBMC from whole blood was conducted as described by Walsh *et al.* [27]. Stimulation of PBMC was performed with phosphate buffered saline (PBS), or a combination of 25 ng/µL phorbol myristate acetate (PMA; Sigma-Aldrich) and 2 mg/mL ionomycin (I; Sigma-Aldrich) for 18 h at 37^o^C in a 5% (v/v) CO_2_ humidified atmosphere. Following stimulation, the cell culture supernatant was collected and stored at −80^o^C. Concentrations of IL-4, IL-6, IL-10, IL-12, TNFα and IFNγ were subsequently determined in these supernatants using porcine-specific cytokine ELISA kits (R&D Systems, Minneapolis, MN) in accordance with the manufacturer's instructions. Samples were analyzed in duplicate on each plate. Duplicate samples with intra-assay precision (CV%) of greater than 10% underwent repeat analysis.

#### 6.2 Isolation and stimulation of lamina propria and intraepithelial lymphocytes and splenocytes and measurement of cytokine production

Lamina propria lymphocytes (LPL) and intraepithelial lymphocytes (IEL) were isolated from porcine ileum tissue samples collected at sacrifice on day 35, as described for human LPL and IEL [28]. For isolation of splenocytes, ∼10 g of spleen was pressed through a 50-mesh screen (Sigma-Aldrich). Remaining cells were washed through with HBSS containing 2% heat inactivated fetal bovine serum (HBSS-FBS; Invitrogen, Paisley, UK). The cells were pelleted at 200×g for 10 min and re-suspended in HBSS-FBS. Erythrocytes were lysed with lysing buffer (BD Biosciences, Devon, UK) according to manufacturer's instructions. Cells were pelleted at 200 × g for 10 min and re-suspended in HBSS-FBS. The cell suspension was filtered through a sterile 70 µm nylon cell strainer (BD Biosciences) and centrifuged for 10 min at 275 × g. The cell pellet was re-suspended in 30% Percoll (Sigma-Aldrich; diluted with 0.9% NaCl) and layered over a 70% Percoll solution. The above gradient separation solution following centrifugation for 20 min at 1230×g yielded a population of mononuclear cells at its interface. Cells were recovered and washed twice in HBSS-FBS by centrifugation at 360×g for 10 min. Both LPL/IEL and splenocytes were counted and re-suspended in complete medium [IMDM + Glutamax (Invitrogen), 20% FBS, 100 U/mL penicillin, 100 µg/mL streptomycin (Invitrogen) at a concentration of 1×10^6^ cells/mL and dispensed into 24 well plates (Sarstedt, Numbrecht, Germany). Stimulation of LPL, IEL and splenocytes was performed and the cell supernatants collected and stored as outlined above for PBMC. Concentrations of IL-4, IL-6, IL-8, and TNFα were subsequently determined in the cell supernatants using multiplex porcine-specific cytokine ELISA kits (Meso Scale Discovery, Gaithersburg, Maryland) in accordance with the manufacturer's instructions. Samples were analyzed in duplicate on each plate. Duplicate samples with intra-assay precision (CV%) of greater than 10% underwent repeat analysis.

#### 6.3 Immune cell phenotyping

Following stimulation, LPL/IEL and splenocytes were resuspended at ∼1×10^6^ cells/mL in PBS containing 2% FBS (PBS-FBS). Primary and secondary antibodies were added at concentrations determined by titration and incubated in the dark at room temperature for 15 min. Cells were washed and re-suspended in PBS-FBS and acquired using a BD FACSCanto II^TM^ flow cytometer. Antibodies used included anti-porcine CD3 PE/Cy5 (Abcam, Cambridge, UK), anti-porcine CD4 fluorescein isothiocyanate (FITC), anti-porcine CD8 phycoerythrin (PE), anti-porcine macrophage FITC (Abd Serotec, Kidlington, UK), anti-porcine CD3 (Abcam), anti-mouse IgG1 peridinin chorolphyll protein (PerCP; Santa Cruz Biotechnology, Santa Cruz, CA), anti-porcine CD45 (Abd Serotec), anti-porcine B cell marker PE (Abcam), anti-mouse IgG1 allophycocyanin (APC), anti-porcine γδ T cell, anti-rat IgG2a APC, anti-rat CD16/CD32 and anti-mouse CD32 (all antibodies were obtained from BD Biosciences, Devon, UK unless otherwise stated). Antibodies were used according to manufacturer's recommendations. The percentages of T and B lymphocytes and macrophages were calculated on leukocyte (CD45^+^) gate, whereas CD4^+^, CD8^+^, CD4^+^/CD8^+^ and γδ T cell subsets were calculated on CD3^+^ gate. At least 50,000 events were acquired and analyzed. Data was analyzed using FACSDIVA software (BD Biosciences).

#### 6.4 Cry1Ab-specific antibody response

For detection of Cry1Ab specific IgA and IgG in pig plasma, 96-well plates were coated overnight at 4^o^C with 1 µg/mL of purified Cry1Ab toxin in 0.05 M carbonate-bicarbonate buffer (pH 9.6). Plates were blocked for 1 h at 37^o^C with 0.01 M phosphate buffered saline (PBS), pH 7.4, containing 1% gelatine (Sigma-Aldrich). PBS alone or serial dilutions (from 1∶5 to 1∶640) of pig plasma from GM maize, non-GM maize or non-maize (control) exposed pigs were added to the plate and incubated for 1 h at 37^o^C. Plates were then incubated with horse radish peroxidase (HRP)-labelled anti-pig IgA or IgG (1∶5000; Bethyl laboratories, Montgomery, TX) antibodies for 1 h at 37^o^C followed by the addition of H_2_O_2_/o-phenylenediamine containing PPD Fast substrate solution pH 5 (Sigma-Aldrich). The plates were developed for 5 min in the dark and the enzyme reaction was stopped by the addition of 4 M H_2_SO_4_. The absorbance was read at 492/630 nm (Dynatech MR 7000, Dynatech Laboratories Ltd., UK). Plasma from non-GM maize exposed pigs was used as a negative control. Plasma from non-maize fed pigs was used as a control to ensure no cross-reactivity with other maize proteins. Samples were analyzed in duplicate and the washing procedure repeated after each step involved manually washing the plates twice with PBS containing 1% gelatine and decanting.

### 7. Tracking of the Cry1Ab protein and gene in feed and porcine digesta, organs and blood

#### 7.1 Cry1Ab protein quantification

Digesta samples were centrifuged for 15 min at 540×g and 10 µL of 10 mM phenylmethylsulfonyl fluoride (PMSF) was added per mL of supernatant and samples were centrifuged for 20 min at 9390×g. Ten microlitres of 10 mM PMSF and 10 µL of 1% sodium azide were added per mL of supernatant, followed by 50 µL of bovine serum albumin (BSA) 15 min later. The samples were centrifuged for 20 min at 9390×g and the resultant supernatant was analyzed for the Cry1Ab protein using a QuantiPlate kit for Cry1Ab/Cry1Ac (Envirologix, Maines, USA) according to the manufacturer's instructions.

#### 7.2 DNA extraction

##### Feed and digesta samples

Milled feed or digesta (250 mg) was incubated in 1000 µL TRIS-EDTA-SDS extraction buffer (pH 8.0) for 1 h at 65°C. Following incubation, the suspension was cooled and 60 mg polyvinylpyrrolidone (PVP) and 500 µL 7.5 M ammonium acetate were added. This was incubated for 30 min on ice followed by centrifugation for 10 min at 14430×g. The supernatant was collected, combined with 1 mL isopropanol and incubated for 30 min on ice. Samples were centrifuged (10 min at 14430×g) and the supernatant was discarded. The remaining DNA pellet was washed with 70% ethanol and was resuspended in 50 µL TE buffer.

##### Animal tissue

DNA was extracted from animal tissue as described by Meyer *et al.* [29]. The supernatant (500 µL) was purified using the Wizard® DNA Clean-up system (Promega, Madison, WI) according to the manufacturer's instructions. Total DNA was quantified using a spectrophotometer (UV-1601 spectrophotometer, Shimadzu) at OD_260 nm_ and purity was assessed by determining the OD_260 nm_:OD_280 nm_.

##### White blood cells

DNA was extracted from 10 µL of buffy coat isolated from blood samples using an Extract-N-Amp Blood PCR kit (Sigma-Aldrich) according to the manufacturer's instructions.

#### 7.3 PCR

A preliminary cross-dilution assay was performed to determine the detection limit of the *cry1Ab-*specific PCR and the possible inhibitory effect of porcine DNA. Three primer pairs targeting an endogenous maize gene, the *cry1Ab* gene and a porcine growth hormone gene, respectively (Table 2) were obtained from Invitrogen (Paisley, UK). Two microlitres of extracted DNA was used in PCR amplifications which were performed in a final volume of 50 µL. Each PCR reaction contained 25 µL of either REDTaq ReadyMix PCR Reaction Mix containing MgCl_2_ (Sigma-Aldrich) (for white blood cells) or DreamTaq Green PCR Master Mix (Fermentas, Ontario, Canada) (for tissue samples and digesta), as well as 0.01, 0.004 or 0.006 µM of the *SW*, *cry1Ab* or *Sh2* primers, respectively and 2 µL of extracted DNA. PCR reactions were performed in a GeneAmp 2400 or 2700 thermal cycler (Applied Biosystems, Foster City, CA). The PCR conditions used are outlined in Table 3. Each set of PCR reactions included a positive control (DNA from MON810 maize), DNA from isogenic non-GM maize, contamination controls without template DNA, and a negative extraction control (DNA from normal pig meat). PCR products were analyzed on 10% polyacrylamide gels run at 200 V for 50 min and visualized by SYBR Green-staining.

**Table 2:** Primers used in PCR reactions for the detection of three target sequences in porcine organ, white blood cell and digesta samples.

Primer name	Sequence (5′-3′)	Specificity	Target gene	Amplicon size (bp)	PCR conditions^1^	Ref.
*Sh2* – F	TTC GGG AGG CAA GTG TGA TTT CG	Plant (endogenous)	ADP glucose pyrophosphorylase	213	94°C×3 min	Jennings *et al*., 2003
					94°C×30 s	
					55°C×30 s	
					72°C×45 s	
					72°C×5 min	
*Sh2* – R	GTC GGC AAG AAT GGA GCA ATT C				94°C×3 min	
					94°C×30 s	
					55°C×30 s	
					72°C×45 s	
					72°C×5 min	
*cry1Ab* - F	CCT GGA GCG CGT CTG GGG CCC TGA TTC T	Plant (transgenic)	*cry1Ab*	211	94°C×3 min	Jennings *et al*., 2003
					95°C×30 s	
					64°C×30 s	
					72°C×30 s	
					72°C×5 min	
*cry1Ab* - R	GGC GCT GCC CCT GAA GCT ACC GTC GAA GTT CT				94°C×3 min	
					95°C×30 s	
					64°C×30 s	
					72°C×30 s	
					72°C×5 min	
*SW* - F	TCA GTT TAC ACT CAC CTG ATA GCA TCT	Animal (porcine)	Pig growth hormone	108	94°C×1 min	Meyer *et al*., 1994
					94°C×30 s	
					60°C×30 s	
					72°C×40 s	
					72°C×3 min.	
*SW* - R	GGG TGG TGG AGA GGG GTG AAT T				94°C×1 min	
					94°C×30 s	
					60°C×30 s	
					72°C×40 s	
					72°C×3 min.	

1 PCR conditions for *Sh*-F & R included 1 cycle at 94°C for 3 min, 30 cycles of 94°C for 30 s, down to 55°C for 30 s and back up to 72°C for 45 s and 1 cycle of 72°C for 5 min; *cry1Ab-*F & R; 1 cycle at 94°C for 3 min, 32 cycles of 95°C for 30 s, down to 64°C for 30 s and back up to 72°C for 30 s and 1 cycle of 72°C for 5 min; *SW*-F & R; 1 cycle at 94°C for 1 min, 30 cycles of 94°C for 30 s, down to 60°C for 30 s and back up to 72°C for 40 s and 1 cycle of 72°C for 3 min.

**Table 3:** The effects of feeding GM maize or non-GM maize with or without mitogenic stimulation on cytokine production from porcine isolated peripheral blood mononuclear cells (**Expt. 1**), splenocytes and lamina propria and intraepithelial lymphocytes (**Expt. 2**) 1 2.

Treatments	Non-GM maize		GM-Maize			*P*		
Cytokines, pg/mL	- PMA/I^3^	+ PMA/I	- PMA/I	+ PMA/I	SEM	trt	PMA/I	trt×PMA/I
*Peripheral blood mononuclear cells* 4								
IL-10	9.6	89.8	12.7	106.5	7.6	0.17	0.001	0.29
IL-12	55.8	1292.3	9.0	333.4	308.5	0.07	0.03	0.09
IL-6	15.9	47.6	7.5	40.3	9.6	0.40	0.01	0.94
IL-4	46.3	301.9	9.7	181.4	75.3	0.24	0.02	0.49
TNFα	0	5586.1	0	3686.9	1546.7	0.26	0.01	0.28
IFNγ	555.9	3041.3	247.7	850.8	644.5	0.04	0.04	0.08
*Splenocytes* 5								
IL-8	283.4	1144.3	67.1	1103.1	245.9	0.60	0.001	0.72
IL-6	1.5	4.2	2.7	6.4	0.8	0.04	0.001	0.56
IL-4	3.9	10.1	11.7	21.2	3.3	0.01	0.02	0.60
TNFα	22.3	274.0	3.3	383.3	41.7	0.28	0.001	0.12
*Lamina propria & intraepithelial lymphocytes* 5								
IL-8	7.6	43.8	12.3	82.2	10.8	0.05	0.001	0.13
IL-6	1.6	3.4	1.5	3.9	0.5	0.69	0.001	0.60
IL-4	2.7	2.1	13.2	21.9	3.3	0.001	0.22	0.17
TNFα	1.1	128.8	1.1	197.2	22.2	0.14	0.001	0.12

1 *n* = 10 pigs in control group and 10 pigs in the GM maize-fed group.

2 Peripheral blood mononuclear cells (PBMC) were isolated from pigs fed treatments for 29 days (**Expt. 1**) and splenocytes and lamina propria & intraepithelial lymphocytes were isolated from pigs fed treatments for 35 days (**Expt. 2**).

3 All isolated cells were stimulated with PBS (- PMA/I) or 25 ng/µL phorbol myristate acetate and 2 mg/mL ionomycin (+ PMA/I) for 18 h at 37^o^C in a 5% (v/v) CO_2_ humidified atmosphere.

4 Data analysis was performed using two factor analysis of variance (ANOVA) with interactions to determine if differences in cytokine production varied by treatment and mitogen stimulation. Baseline values measured on day 0 were included as covariates in the model.

5 Data analysis was performed using two factor analysis of variance (ANOVA) with interactions to determine if differences in cytokine production varied by treatment and mitogen stimulation.

### 8. Statistical analysis

All data were analyzed as a complete randomized block design using the GLM procedures of SAS [30]. For all response criteria, the individual pig was the experimental unit. Treatment effect was tested against residual error term with initial bodyweight as a blocking factor. Growth performance data were analyzed as a one-factor analysis of variance (ANOVA) using the GLM procedure of SAS. Cytokine production data was analyzed as a two-factor ANOVA with interactions to determine if differences in cytokine production varied with diet and mitogenic stimulation. Baseline cytokine levels (day 0) were also used as covariates in the model. The level of significance for all tests was *P*<0.05. Trends were reported up to *P* = 0.10.

## Results

### 1. Analysis of GM and non-GM maize for the *cry1Ab* gene, mycotoxins and pesticide residues

**Figure 1:**
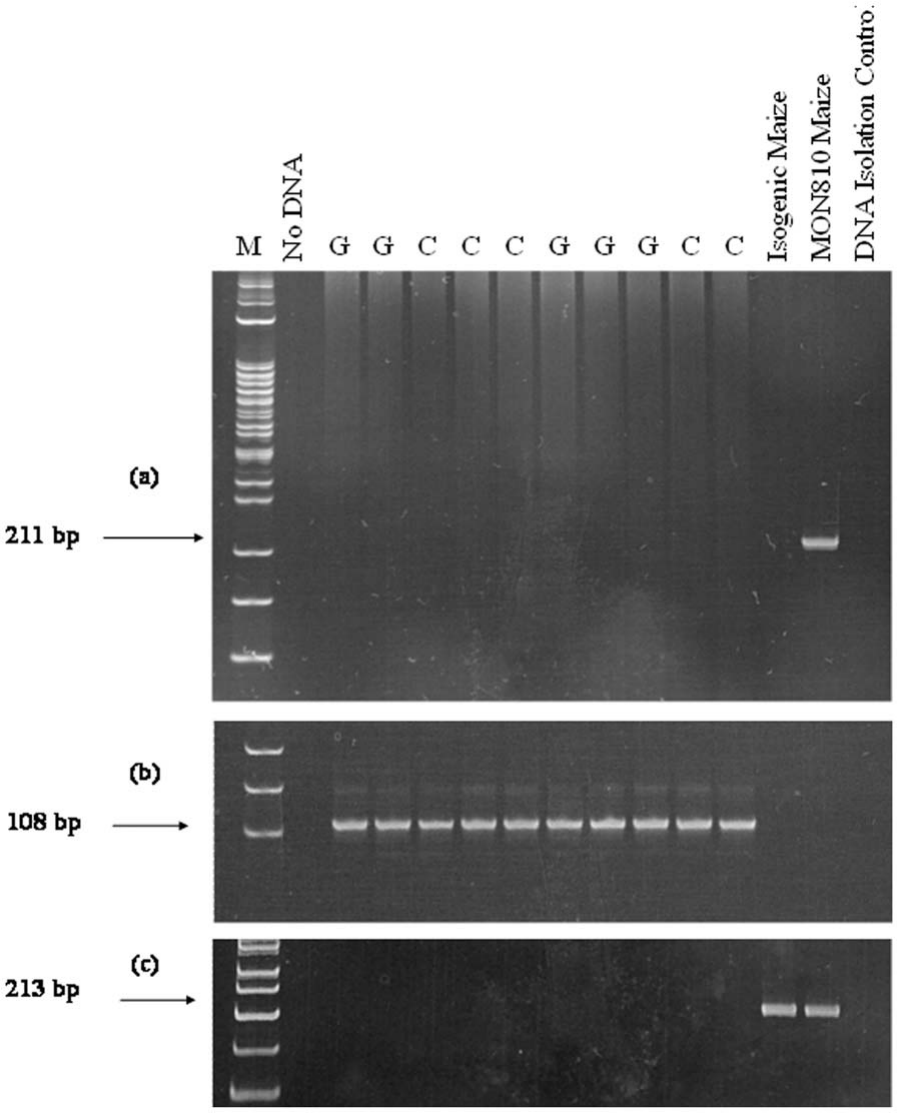
Fate of transgenic DNA from orally administered Bt MON810 maize in pigs. PCR products from liver samples of 10 pigs (**Expt. 1)** fed either GM maize (G) or control maize (C), for each of the target gene fragments; (a) *cry1Ab*, (b) *SW* (endogenous porcine) and (C) *Sh2* (endogenous maize). Arrows indicate the expected size of PCR products. Reactions without template DNA (no DNA) and with purified genomic DNA from MON810 maize and isogenic parent line maize were included as controls. A DNA isolation control which did not contain any sample material was also used. M = molecular weight marker.

The GM maize was found to have >5% event-specific *cry1Ab* gene insert. However, the non-GM maize was also found to have 0.20% event specific *cry1Ab* gene insert. The *cry1Ab* gene was also detected in the GM maize but was not found in the non-GM maize analyzed (Fig. 1) which indicates that the level of *cry1Ab* gene contamination of the non-GM maize was too low to be detected by non-quantitative PCR. Conventional feed ingredients containing unintentional traces of genetically modified organisms below a threshold level of 0.9% of total DNA are not required to be labelled as GM [19]. The levels of all mycotoxins detected in the GM and non-GM maize were below the maximum allowable levels as, outlined in EU legislation (Commission Regulation (EC) No 576/2006). The GM and non-GM maize were also negative for all pesticide residues tested.

### 2. Expt.1. Effect of short-term feeding of GM maize on the systemic immune response

**Figure 2:**
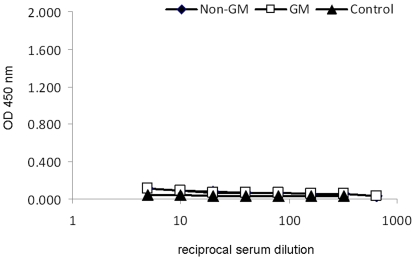
Effect of feeding non-GM maize (♦), GM maize (□) or non-maize feed (▴) to pigs for 31 days on plasma concentration of Cry1Ab specific IgG antibody (Expt. 1). Cry1Ab specific IgG antibody was not detected in any of the samples assayed.

**Figure 3:**
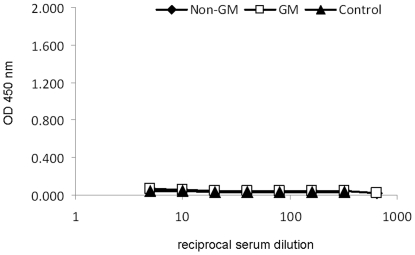
Effect of feeding non-GM maize (♦), GM maize (□) or non-maize feed (▴) to pigs for 31 days on plasma concentration of Cry1Ab specific IgA antibody (Expt. 1). Cry1Ab specific IgA antibody was not detected in any of the samples assayed.

Mitogenic stimulation resulted in a significant (*P*<0.05) increase in the production of IL-10, IL-6, IL-4 and TNFα by PBMC (Table 3). There was a tendency for a treatment×PMA/I interaction for IL-12 (*P* = 0.09) and IFNγ (*P* = 0.08) production from isolated PBMC. Both IL-12 and IFNγ production by isolated PBMC tended to be reduced following PMA/I stimulation in pigs fed GM maize compared to non-GM maize-fed control pigs following 29 days of feeding. In addition, Cry1Ab-specific IgG (Fig. 2) or IgA (Fig. 3) were not detected in plasma taken from pigs fed either the GM or non-GM maize or non-maize feed (control) even at the lowest dilution used.

### 3. Detection of transgenic and endogenous plant genes in white blood cells, tissues and digesta of GM maize-fed pigs

Neither endogenous (*Sh2*) nor transgenic (*cry1Ab*) plant gene fragments were detected in the white blood cells or any of the tissues examined (Table 4). However, as expected all the white blood cells and tissue samples were positive for the endogenous (*SW*) porcine gene. PCR analysis of liver samples is shown in Figure 1 as an example. The endogenous porcine gene was also detected at a relatively high frequency in digesta along the length of the GIT in both the non-GM maize and GM maize-fed pigs (stomach, 100%; ileum, 80 and 70%; cecum, 60 and 70%; colon, 100 and 90%, respectively). The endogenous plant gene was detected in all stomach digesta samples taken from both the GM and non-GM maize-fed pigs, while the *cry1Ab* gene was only detected in the stomach digesta of pigs fed the GM maize diets (Table 5). Further down the GIT, the endogenous maize gene was detected in the ileal digesta of 50% of the non-GM maize-fed pigs and 20% of the GM maize-fed pigs, in the cecal digesta of 10% of each treatment group and was undetectable in colon samples. The *cry1Ab* gene was also detected in the lower gastrointestinal tract of GM maize-fed pigs, but only in the ileal and cecal digesta (20% and 10%, respectively) and at a lower frequency than in the stomach digesta. The *cry1Ab* gene was not detected in colon samples from either GM maize or non-GM maize-fed pigs.

**Table 4:** Detection of endogenous maize and porcine genes and transgenic *cry1Ab* gene in tissue and white blood cells of pigs fed GM maize versus pigs fed a non-GM maize diet for 31 days (**Expt. 1**)^1^.

Fragment amplified	Organ Tissue											
	Heart		Liver		Spleen		Kidney		Muscle		White blood cells	
GM treatment^2^	-	+	-	+	-	+	-	+	-	+	-	+
**Endogenous**												
*Sh2* (maize)	0	0	0	0	0	0	0	0	0	0	0	0
*SW* (porcine)	10	10	10	10	10	10	10	10	10	10	10	10
**Transgenic**												
*cry1Ab* (maize)	0	0	0	0	0	0	0	0	0	0	0	0

1 Number of samples that tested positive for the gene of interest out of 10 samples analyzed. One sample was tested per pig (*n*  = 10 pigs per treatment).

2 GM treatments; - denotes non-GM maize-fed pigs and + denotes GM maize-fed pigs.

**Table 5:** Detection of endogenous maize and porcine genes and transgenic *cry1Ab* gene in stomach, ileal and cecal digesta and colon samples of pigs fed GM maize versus pigs fed a non-GM maize diet for 31 days (**Expt. 1**)^1^.

Fragment amplified	Digesta							
	Stomach		Ileum		Cecum		Colon	
GM treatment^2^	-	+	-	+	-	+	-	+
**Endogenous**								
*Sh2* (maize)	10	10	5	2	1	1	0	0
*SW* (porcine)	10	10	8	7	6	7	10	9
**Transgenic**								
*cry1Ab* (maize)	0	10	0	2	0	1	0	0

1 Number of samples that tested positive for the gene of interest out of 10 samples analyzed. One sample was tested per pig (*n*  = 10 pigs per treatment).

2 GM treatments; - denotes non-GM maize-fed pigs and + denotes GM maize-fed pigs.

### 4. Transgenic Cry1Ab protein detection in plasma, tissue and digesta of GM maize-fed pigs

The Cry1Ab protein was not detected in the heart, liver, kidney, spleen, muscle or plasma of pigs fed either GM maize or non-GM maize diets (Table 6). Likewise, no transgenic protein was detected in the stomach, ileum, cecum or colon digesta of pigs fed non-GM maize diets. The Cry1Ab protein was, however, detected in the stomach and cecal digesta of 30% of the GM maize-fed pigs 3 h after the last GM maize meal was administered and in the colon and ileal digesta of 80% of these pigs. The concentration of Cry1Ab protein detected in the digesta samples of GM maize-fed pigs was very low and ranged from 2.41 – 2.74 ng/mL.

**Table 6:** Detection of the transgenic Cry1Ab protein in tissue, plasma and gastrointestinal digesta of pigs fed GM and non-GM maize.

	Number of positive samples^1^		Mean concentration (ng/mL)	Positive detection frequency in GM-maize fed pigs^2^
	Non-GM maize	GM Maize		
Heart	0	0	BDL^3^	0
Liver	0	0	BDL	0
Spleen	0	0	BDL	0
Kidney	0	0	BDL	0
Muscle	0	0	BDL	0
Plasma	0	0	BDL	0
Stomach digesta	0	3	2.74	30
Ileal digesta	0	8	2.45	80
Cecum digesta	0	3	2.41	30
Colon digesta	0	8	2.67	80

1 Number of samples that tested positive for the Cry1Ab protein out of 10 samples analyzed. One sample was tested per pig (*n* = 10 pigs per treatment).

2 Percentage of samples positive for the Cry1Ab protein taken from GM maize fed pigs i.e. (number of positive samples/number of samples tested) ×100.

3 BDL = below detectable levels.

### 5. Expt. 2. Effect of feeding GM maize on body weight and growth performance

There was no effect of feeding GM maize to pigs on growth performance or body weight (Table 7).

**Table 7:** The effects of feeding GM or non-GM maize for 35 days on weanling pig growth performance (**Expt. 2**)^1^.

	Non-GM Maize	GM Maize	SE	*P* 2
***Overall, day 0 - 30***				
ADG, g/d	321	355	37.5	0.53
ADFI, g/d	465	479	41.8	0.82
FCE	1.54	1.43	0.097	0.45
day 30 BW, kg	17.6	18.6	1.21	0.54

ADG: average daily gain, ADFI; average daily feed intake, FCE; feed conversion efficiency = feed intake (g)/body weight (g), BW; body weight.

1 *n* = 12 pigs in control group and 11 pigs in the GM maize-fed group.

2 Data analysis was performed using one factor analysis of variance (ANOVA) using the GLM procedure of SAS.

### 6. Effect of short-term feeding of GM maize on the local immune response

**Figure 4:**
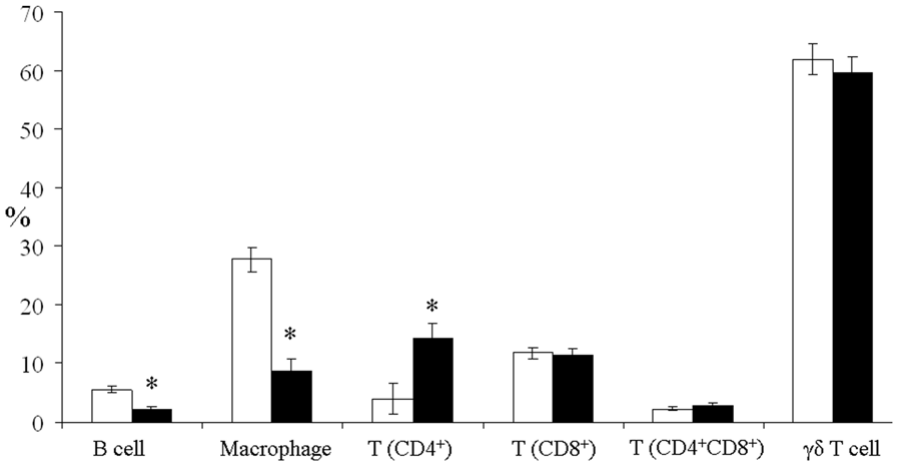
Changes in the proportion of different leukocyte populations in the ileum of pigs fed non-GM (□) or GM (▪) maize for a period of 35 days (Expt. 2). Mean values±SEM were calculated as a percentage of the total lamina propria and intra-epithelial lymphocyte populations. * Significance at *P*<0.01.

**Figure 5:**
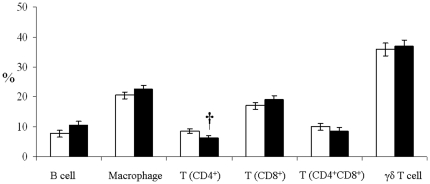
Changes in the proportion of different leukocytes populations in the spleen of pigs fed non-GM (□) or GM (▪) maize for a period of 35 days (Expt. 2). Mean values±SEM were calculated as a percentage of the total splenocyte population. †Tendency for a significance at *P*<0.10.

Short-term feeding of GM maize to pigs resulted in increased IL-6 and IL-4 production from isolated splenocytes (*P*<0.05) and increased IL-8 and IL-4 production from isolated LPL and IEL (*P*<0.05; Table 3) compared to non-GM maize-fed pigs. There was no effect of treatment on TNFα production from splenocytes or LPL/IEL or on IL-8 production from splenocytes or IL-6 production from LPL/IEL. Phorbol myristate acetate/ionomycin stimulation resulted in a significant (*P*<0.05) increase in production of IL-8, IL-6 and TNFα by splenocytes and LPL/IEL while IL-4 production was increased by mitogen stimulation in the spleen but not the ileum. Local immune responses were also assessed by phenotyping leukocytes isolated from spleen and ileum. Short-term feeding of GM maize to pigs resulted in a lower proportion of ileal B cells and macrophages than in non-GM maize-fed pigs (*P* = 0.001; Fig. 4). There was no effect of treatment on the proportion of CD8^+^ T cells, CD4^+^CD8^+^ T cells or γδ T cells isolated from the ileum. However, the proportion of CD4^+^ T cells increased in response to feeding GM maize (*P* = 0.01). The number of CD4^+^ T cells as a proportion of the total splenocyte population tended to decrease (*P* = 0.06; Fig. 5) in response to short-term feeding of GM maize to pigs. There was no effect of feeding GM maize on the numbers of B cells, macrophages, CD8^+^ T cells, double positive CD4^+^CD8^+^ T cells and γδ T cells isolated from the spleen.

## Discussion

Previously, a study with mice reported alterations in both the local and systemic immune systems in response to feeding Bt (MON810) maize for 30 days post-weaning [17]. Several disturbances in lymphocyte subsets at gut and peripheral sites were documented in this study. We observed a similar decrease in CD4^+^ T cells and B cells in the spleen and ileum, respectively, when Bt (MON810) maize was feed to weanling pigs for 35 days post-weaning (Expt. 2). However, an increase in CD4^+^ T cell subsets within the porcine ileal lymphocyte population in Expt. 2 was contrary to findings in mice [17] and unlike the results with mice, we also found a reduction in porcine ileal macrophages in response to feeding Bt (MON810) maize. The implications of the alterations in CD4^+^ T cells, B cells and macrophages in the ileum and CD4^+^ T cell in the spleen have yet to be explained. However, work by Murtaugh *et al*. [31] in pigs found evidence of an anti-proliferative effect of IL-4 on B-cells. Interleukin-4 production from cultured ileal lymphocytes was elevated in GM maize-fed pigs (Expt. 2) and this may account for the observed reduction in B-cells. While GM exposure also increased IL-4 production in the spleen in Expt. 2, there was no effect on B cell populations indicating a potential site-specific effect of IL-4. Previous work found that IL-6 and IL-4 play a major role in mediating B-cell activation and antibody production [32]. Interleukin-6 is also known to antagonize the IL-12/IFNγ mediated differentiation of naïve T cells towards a Th1 inflammatory type response in favor of the Th2 humoral immune response. In Expt. 2, the production of IL-6 and IL-4 from cultured splenocytes and IL-4 from ileal lymphocytes was increased in GM maize-fed pigs and results from Expt. 1 showed a decrease in IL-12 and IFNγ from cultured PBMC. Pro-inflammatory cytokines, IL-6 and IL-4 are known to be involved in allergic and inflammatory responses [32]. However, the increase in antigen-specific IgA and IgG that accompanies a Th2-mediated allergic inflammatory response [33] was notably absent in Expt. 1 which indicates that feeding GM maize did not elicit an allergenic response. Adel-Patient *et al*.[34] also reported finding no specific anti-Cry1Ab antibody in serum from mice given MON810 maize after either i.g or i.p sensitization. The basal concentration of both IL-12 and IFNγ from resting PBMC (no PMA/I stimulation) isolated from non-GM maize-fed pigs was greater than similar cytokine concentrations in GM maize-fed pigs. By nature of the transgene insertion, GM maize is protected from insect damage and may as a result contain less endotoxins than its non-GM maize counterpart. The potentially greater exposure to endotoxins from feeding non-GM maize may account for the elevated Th1 profile of cytokines evident in both resting and stimulated PBMC isolated from pigs fed non-GM maize. Therefore, feeding GM maize to pigs may protect against a systemic inflammatory response characterized by an elevated Th1 cytokine profile.

The increase in cytokine production from cultured cells in Expt. 2, while statistically significant was numerically small and unlikely to be of biological relevance. These findings together with the lack of Cry1Ab-specific antibody production in blood make the development of a Th2-mediated allergic response highly unlikely. Overall, the findings from the two experiments suggest that some GM maize-induced systemic and local immune alterations are occurring in the weaned pig. The Cry1Ab protein, which was found in the majority of small intestinal digesta samples from GM maize-fed pigs (Expt. 1), has been shown to lack homology with known allergens and is not at risk of causing allergenic cross-reactivity [35]. The presence of the Cry1Ab protein in the GIT of GM maize-fed pigs is the only measured difference between these pigs and the control pigs. Therefore, the non-allergenic alterations observed in the immune response of GM maize-fed pigs most likely are attributed to the Cry1Ab protein and in some cases feeding GM maize may prevent a systemic Th1 inflammatory response.

One of the main consumer concerns with the use of GM foods is transfer of the transgenic DNA to human tissues or to animal products such as meat. Numerous animal studies have been conducted in which transgenic DNA has not been detected in food products derived from animals fed GM feed ingredients [20], [21], [22], [36], [37]. However, the transfer of endogenous plant DNA across the gut barrier is a natural phenomenon, as it has been detected in both animal tissue and products [24], [36], [38]. Previously, Sharma *et al*. [23] detected a 278 bp fragment of the *cp4epsps* transgene found in Round-Up Ready canola in the liver and kidney of swine. However, only one liver and one kidney sample out of 36 samples tested were positive for the transgenic DNA fragment. Likewise, a 519 bp fragment of *cry1Ab* was detected in the blood, liver, kidney, and spleen of piglets following 35 days of administration of Bt (MON810) maize [24]. However, the intact *cry1Ab* gene (3500 bp) or the genes smallest functional unit (1800 bp) was never detected. In Expt. 1, the target *cry1Ab* gene fragment (211 bp) was not detected in the white blood cells, heart, liver, spleen, kidney or muscle of pigs fed GM maize for 31 days. While much emphasis has been placed on minimizing potential cross-contamination between animals fed GM feed ingredients and controls in other studies, transgenes have been detected in tissue [23], [24]. However in Expt.1, no cross-contamination occurred and the *cry1Ab* gene was not detected in tissues or blood and this may question the effectiveness of contamination preventative measures used in previous studies where transgenes were found in tissues. Furthermore, findings from Expt. 1 demonstrate a lack of transfer of endogenous plant DNA across the gut barrier into organs and blood. The frequency of gene detection is dependent on copy number of the genes ingested. In Expt. 1, the *Sh2* gene was used as a control to compare the behavior of a single copy gene to that of the single copy *cry1Ab* gene. In some cases, the multiple copy *Rubisco* or *Zein* genes are used as indicators of endogenous plant DNA transfer [24], [36]. These genes have a higher detection frequency than single copy genes such as *Sh2* which may explain the discrepancies between our findings and those that have detected endogenous plant DNA in animal tissue. Similar to previous findings [39], the detection of both the endogenous plant and *cry1Ab* genes in the digesta of GM maize-fed pigs decreased during passage through the GIT from 100% recovery in the stomach to undetectable in the feces. Chowdhury *et al.* [39] found that a primer pair with a 110 bp PCR product detected *cry1Ab* in a greater number of pig digesta samples than a primer pair that detected a 437 bp fragment. Thus, the recombinant DNA appears to be degraded as it passes through the GIT. Consequently, the recombinant DNA may have been present in smaller fragments than were detectable using the *cry1Ab* primer pair used in the Expt. 1 (211 bp) accounting for the low frequency of detection in the distal GIT.

Similar to the findings documented by Yonemochi *et al*. [20], transgenic Cry1Ab protein was not detected in the plasma or organs of any pigs from Expt. 1. This is not surprising, considering that the transgene was also undetectable in plasma and organs. The Cry1Ab protein was only detected in 30% of stomach samples taken from GM maize-fed pigs and at low concentrations (2.74 ng/mL) even though the transgene was recovered from all of these samples. Chowdhury *et al*. [39] reported much higher levels of Cry1Ab protein (300±140 ng/g) in rectal digesta of pigs. However, these pigs were heavier and fed a diet containing 20% more maize than pigs in the current study. Numerous proteolytic cleavage sites for pepsin in particular, have been reported within the Cry1Ab protein [40]. Consequently, the Cry1Ab protein is likely to have undergone some degree of degradation by pepsin accounting from the lower detection frequency in the stomach. The lower detection frequency of the *cry1Ab* gene compared to the protein further down the GIT (small intestine, cecum and colon) found in Expt. 1 has been observed previously [39] when a 437 base pair primer was used for the detection of the *cry1Ab* gene in pig digesta. However, in the same study when a *cry1Ab* primer for a shorter fragment length (110 bp) was used, transgene detection frequency increased and mirrored that of the protein detection rates. Likewise, Einspanier *et al.* [41] found that the use of primers with shorter expected fragment lengths increased the chance of detection of plant and maize DNA from cows. In Expt. 1, the use of a 211 bp primer instead of a primer with a shorter expected fragment length may account for the discrepancies observed in detection frequencies between the *cry1Ab* gene and protein at similar sites along the GIT.

No consistent effects on feed intake and ADG have been reported in the numerous pig-feeding studies that have compared GM maize with conventional maize varieties [42], [43], [44]. Previous work published by our group [26] found that, similar to results from Expt. 2, feeding GM maize to weanling pigs had no effect on growth rate or body weight following 31 days of feeding. Although an increase in feed intake was observed previously [26] in Bt (MON810) maize-fed pigs, feed intake was not affected in Expt. 2. Consequently, short-term feeding of GM maize is unlikely to effect the growth of weanling pigs.

In conclusion, data obtained from short-term feeding of GM maize to weanling pigs have demonstrated no adverse effects on growth performance. Maize-derived DNA, either of intrinsic or recombinant origin, was largely degraded in the GIT. There was no evidence of *cry1Ab* gene or protein translocation to organs or plasma. Transgenic protein was detected in GIT digesta but at very low concentrations. Exposure to GM maize did induce some alterations in localized and peripheral immune responses in weanling pigs which require further investigation. The lack of Cry1Ab specific Ig production in plasma however suggests that the immune response was not allergenic and there is evidence to indicate that feeding GM maize may help to prevent an elevation in the inflammatory Th1 cytokine profile observed following non-GM maize consumption. Although the significance of the alterations in immune response have yet to be established, the lack of recombinant DNA or protein translocation to tissues or changes in growth should help to offer assurance to consumers as to the safety of GM feed ingredients. To further investigate the changes observed in this study, we are currently conducting a study to assess any immune responses that may arise from long-term feeding of GM maize to pigs.
